# Power Tower Inspection Simultaneous Localization and Mapping: A Monocular Semantic Positioning Approach for UAV Transmission Tower Inspection

**DOI:** 10.3390/s22197360

**Published:** 2022-09-28

**Authors:** Zhiying Liu, Xiren Miao, Zhiqiang Xie, Hao Jiang, Jing Chen

**Affiliations:** College of Electrical Engineering and Automation, Fuzhou University, Fuzhou 350108, China

**Keywords:** power lines inspection, UAV inspection, visual positioning, simultaneous localization and mapping, semantic SLAM

## Abstract

Realizing autonomous unmanned aerial vehicle (UAV) inspection is of great significance for power line maintenance. This paper introduces a scheme of using the structure of a tower to realize visual geographical positioning of UAV for tower inspection and presents a monocular semantic simultaneous localization and mapping (SLAM) framework termed PTI-SLAM (power tower inspection SLAM) to cope with the challenge of a tower inspection scene. The proposed scheme utilizes prior knowledge of tower component geolocation and regards geographical positioning as the estimation of transformation between SLAM and the geographic coordinates. To accomplish the robust positioning and semi-dense semantic mapping with limited computing power, PTI-SLAM combines the feature-based SLAM method with a fusion-based direct method and conveys a loosely coupled architecture of a semantic task and a SLAM task. The fusion-based direct method is specially designed to overcome the fragility of the direct method against adverse conditions concerning the inspection scene. Experiment results show that PTI-SLAM inherits the robustness advantage of the feature-based method and the semi-dense mapping ability of the direct method and achieves decimeter-level real-time positioning in the airborne system. The experiment concerning geographical positioning indicates more competitive accuracy compared to the previous visual approach and artificial UAV operating, demonstrating the potential of PTI-SLAM.

## 1. Introduction

Regular inspection of overhead transmission lines is of great significance to secure uninterrupted distribution of electricity [1]. The traditional methodology for inspecting overhead transmission lines has been typically carried out using foot patrol [1], which is famous for its low efficiency, labor intensiveness and hostile work environment. To remedy these deficiencies, unmanned aerial vehicles (UAV) have been widely used in transmission line inspection and are expected to replace foot patrol due to their higher efficiency, lower cost and higher trafficability; this has made autonomous UAV inspection a hot issue worldwide [1].

### 1.1. Automatic UAV Power Line Inspection

**Figure 1:**
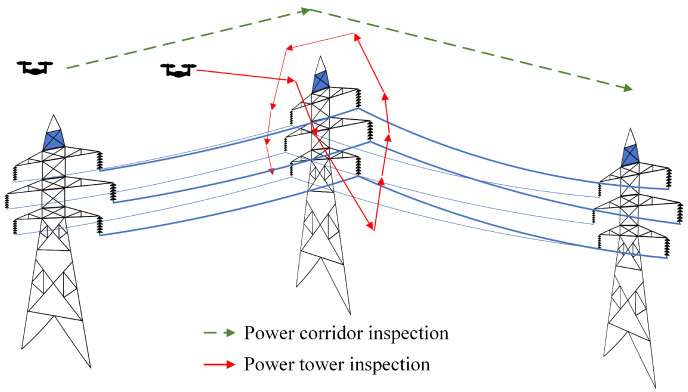
Diagram of power line inspection.

Most studies of UAV autonomous inspection fall into the category of power corridor inspection [2], which can be directly guided by power lines [3,4,5,6] and towers [7], as shown in Figure 1. To realize autonomous inspection for power towers, the industrial world, including Chinese utility companies and their main inspection service providers (e.g., DJI enterprise), adopt a strategy of geographical waypoint tracking. In a typical waypoint-tracking-based automatic inspection, the patrol team carries the UAV to the tower and sets up the inspection task, including the global positioning system (GPS) coordinate and orientation of UAV for each checkpoint. The UAV automatically flies to the designated position, adjusts its attitude and collects inspection data. There are two ways to obtain the GPS waypoints and their shooting attitude: geometrically calculating in a three-dimensional surveying and mapping map [2], or recording the flight path of a skilled inspector. Such a strategy relies highly on high-precision positioning equipment such as real-time kinematic (RTK) fixed GPS. When the RTK signal coverage is not stable, manual operating is unavoidable.

To overcome this limitation, Alvaro et al. [8] developed a cooperative positioning architecture with an unmanned ground vehicle (UGV) and a UAV for tower inspection. In this architecture, both UGVs and UAVs are equipped with RTK, while the UGV carries an additional visual positioning identifier to provide a positioning gain for the UAV. Nonetheless, the positioning is entirely unrelated to the scene so that the RTK fixed GPS is required to perform pre-programmed track flight and the bulky identifier also limits its application. Owing to the lack of real-time environmental information, the existing positioning approaches cannot support further intelligent tasks such as autonomous navigation and self-adapting to scene changes (e.g., vegetation invasion to the flight route and location movement of inspection target caused by maintenance).

### 1.2. Scene Challenges for Monocular Object SLAM

In the context of robotics, a study of surrounding perception often refers to semantic simultaneous localization and mapping (SLAM) [9]. Considering the hardware compatibility concerning inspecting UAVs, the monocular semantic SLAM is a stronger candidate for a power line inspection scenario; however, it faces changes in the power tower inspection scene.

The semantic SLAM concerning navigation usually utilizes objects as the landmarks for locating the scene map and providing navigation information, i.e., the object SLAM. There are two main approaches to extract and represent spatial information of objects: using preset templates to estimate the position and orientation of objects from sparse environment mapping [10] and using dense mapping to reconstruct objects [11]. Concerning the former, the structure and appearance of power tower components are typically variable. For example, the insulator string, which is an important component used to hang the power lines, has a very variable aspect ratio (possibly from 0.1 to 1.2) and is likely to be deformed by the traction of conductor. Fitting such objects requires the template to be very adaptable. When using Quadric to fit the objects (like QuadricSLAM [12]), such a large aspect ratio can cause even a slight error to lead to a huge deviation. Cube-SLAM [13] utilizes a 2D bounding box and the vanishing points of the surface line to model the objects as a cube; however, the sufficiently linear edges are not always present on the tower components like insulator string.

As for using dense mapping to reconstruct objects, limited by the computation consumption and real-time requirement, it is usually achieved by the direct method-based SLAM [11], which relies on the assumption of brightness constancy across multiple images [14] and is violated when illumination varies. Power tower inspection needs to observe objects from multiple angles in outdoor bright light and in scenarios where illumination changes often occur. In addition, power tower components are typically made of metal, ceramic or glass and the surface reflection easily causes a dramatic change of local brightness, resulting in the failure of direct method pixel tracking.

### 1.3. The Objective of the Paper

Motivated by the observations above, this paper introduces an idea of using the structure of a power tower to realize visual positioning of UAVs for tower inspection. In order to select the semantic objects from hundreds of tower components, we analyze the characteristics of tower inspection and offer recommendations concerning the aspects of visual detectability and scene structure describing ability. On this basis, a geographical UAV positioning scheme using prior knowledge of semantic objects is devised. A monocular semantic SLAM combining the feature-based method SLAM and the fusion-based direct method, termed power tower inspection SLAM (PTI-SLAM), is developed to perform robust positioning and semi-dense object mapping under the challenges of a tower inspection scene. The fusion-based direct method is designed to work against complex illumination by using a sliding window strategy to transform long-term pixel tracking into batch-based semantic observation fusion. PTI-SLAM utilizes loose coupling between the SLAM task and the semantics task to guarantee a real-time performance in a UAV onboard platform and further prevent the vulnerability of the direct method from affecting SLAM positioning.

## 2. Semantics Selection and Geographical UAV Positioning Scheme

### 2.1. Semantic Object Selection

For a vision task, the suitability of a semantic object can be considered from two perspectives: the capability of the scene structure representation and the adaptability for visual detection.

#### 2.1.1. Structure Semantics for Inspection Flight

With the aim of examining the tower meticulously at a close range, the UAV needs to traverse the tower and hover from time to time to perform more detailed observation and data acquisition. The flight trajectories, including route and hovering points, are carefully charted to match the architecture of the tower and the assembly of power lines, resulting in a variety of flight scheme. Still, the guidelines for a flight scheme can be well tracked by the intent of each decomposed movement.

**Figure 2:**
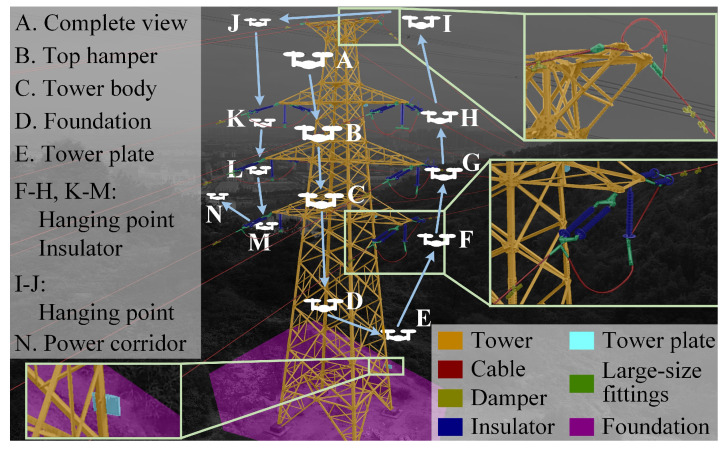
UAV proximity transmission tower inspection diagram.

Figure 2 shows a typical flight scheme adopted by a local utility company for a double circuit line tower inspection. The tower is dissected according to the component categorization performed by the China Electric Power Research Institute (CEPRI) during power line inspection aerial image processing competitions. The large-size fittings involve a variety of accessories such as yoke plate and clamp. The hovering points A–N are the checkpoints directed by the examination targets which are listed on the left side of Figure 2. The inspection flight is essentially a traversal of the checkpoints outside the no-fly zone, which is defined by the outermost edged of the power corridor and the regulations about safety distance (typically 2.5 m).

The insulator string is the examination target of hovering points F–H and K–M, while I and J can be located using a clamp and a damper. The particularity of these points is that they are usually located in the outermost areas of the tower and delineate the outline of the power corridor, which is crucial to the identification of a no-fly zone. The targets of hovering points B and C are the structures of the tower. Mapping and modelling such large-size hollowed-out structures requires multi-angle observations, which are usually only possible after a tower has been totally inspected. This means they cannot be used for cognition scenes and navigation during proximity inspection. Besides that, the reconstruction of a tower is less important since checkpoints F–M can also outline the tower. The targets of checkpoints A and N are not the concrete objects, they are directed by the semantic structure of the scene.

#### 2.1.2. Component Detectability

In terms of visual detection adaptability, object detection is taken as representative of a semantic detection task since it attracts more attention compared to the other methods such as semantic segmentation and image classification and makes an efficient comparison more easily. We summarize reports of the last decade and list the best detection results in the ‘Object Detection’ column of Table 1. The components are classified similarly to Figure 2. Similar to the large-size fittings, the small-size fittings involve a variety of accessories including bolts, core-pin and other tiny accessories. The ancillary facilities consist of the support units like lightning arrester, nameplate and anti-bird tool. The statistics on the foundation and small fittings are marked with the ‘/’ symbol, as their studies are too outdated or too rare to make an efficient comparison. The detections for insulator and damper achieved the most outstanding result in the laboratory, with average precision (AP) of over 95%. Such a high adaptability to visual detection benefits from the conspicuousness in the aerial image and the unified structural features. Although the cable and tower have similar precision in detection, they are hardly helpful in proximity inspection. Zhai et al. [15] detect multiple fittings using hybrid prior knowledge and achieve mean AP (mAP) of 78.6%, where the mAP of the clamp is 78.86%. Kong et al. [16] evaluate the detection performance on the tower plate and obtain an AP of 73.2%. They are among the best results in related studies, while there is still a big gap compared to the detection of the insulator and the damper. The main reason for the low accuracy is that the components are usually small and easily occlude each other.

The results of two nationwide invitational competitions organized by CEPRI are also taken into account. The two competitions are aimed at detecting defects of components in the aerial images. Their datasets are collected by UAVs and helicopters, separately, and labelled with CEPRI. Although there is a significant difference between defect detection and object detection, they are valuable as they provide fairness comparison based on an authority dataset. In addition, the adaptability of defect detection may let fall a further hint concerning the object detection performance of the components with defects. Among all the components, the insulator shows a high adaptability to defect detection and performs consistently in both competitions. In UAV datasets, the damper is categorised as large-size fittings and contributes the majority of the experiment results for this category. Apparently it has a similar summing-up to the insulator.

To summarize, the insulator and damper are the stronger semantics candidates for navigation purposes as they are highly associated with the inspection target and have significant advantages in terms of scene structure-describing ability and visual detection adaptability. Taking tower plates and the large-size fittings such as clamps as semantic targets has a certain risk of failure since the visual detection is not always reliable. Although the foundation is one of the inspection targets, its visual detectability has not yet been proved. In addition, the foundation is easily obscured by the tower body in aerial view, making accurate positioning difficult. Therefore, it is not recommended as a semantic object. In this paper, the insulator is selected as semantics representatives for subsequent work.

### 2.2. Geographical UAV Positioning Scheme

The key to the applying visual positioning in navigation is to establish an association between UAV positioning and flight tasks. Semantic objects offer an opportunity to accomplish this. However, some special checkpoint targets are not concrete objects (e.g., checkpoint N) or are not suitable for visual detection (e.g., checkpoint D), making location recognition difficult.

Alternatively, we devise an object-based geographical UAV positioning strategy to achieve geolocation without relying on on-board GPS. Specifically, we utilize the existing tower surveying and mapping data to take the geographical location of the semantic object as prior knowledge. The transformation relationship between the visual positioning coordinates and geographical coordinates is obtained by using object localization in the semantic map established by semantic SLAM. Such a transformation makes it possible to achieve a real-time geographical UAV positioning and use visual positioning instead of RTK-fixed GPS in the waypoint flight. In addition, since the UAV position is calculated based on object location, this strategy can accommodate a certain degree of scene change.

## 3. Methodology

### 3.1. Framework of PTI-SLAM

**Figure 3:**
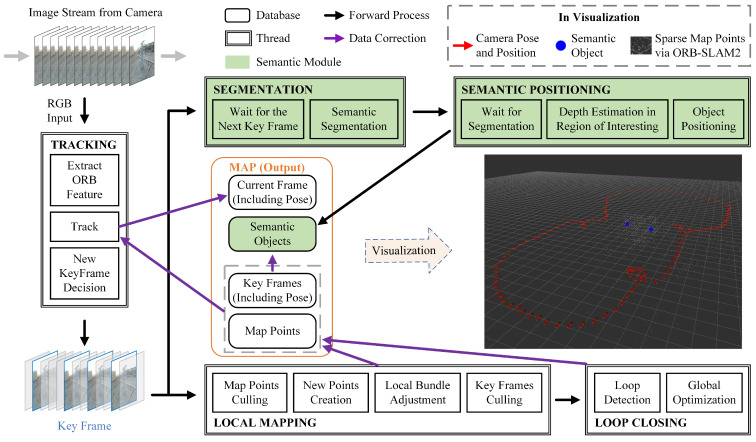
The framework of PTI-SLAM.

The proposed PTI-SLAM is built on a famous open source framework termed DS-SLAM [20], which improves the RGB-D mode of ORB-SLAM2 for the indoor dynamic environment by segmenting and removing dynamic objects. A general overview of PTI-SLAM is shown in Figure 3. There are five threads run in PTI-SLAM: tracking, local mapping, loop closing, segmentation and semantic positioning, where the white boxes are the modules from OBR-SLAM2; the green boxes are the semantics modules designed for power line component positioning. The RGB image captured by the pan-tilt camera is first fed into the tracking thread. The tracking thread estimates the camera pose and position with every frame by matching the ORB feature to the local map and minimizing the reprojection error. The keyframes are the representations extracted from the frames. The generation of keyframes is determined according to the keyframe interval, the tracking status, the local thread occupancy and the number of map points. Subsequently, the keyframes are served to two parallel backends for two separate tasks.

The first task is to generate and maintain the sparse map points and participate in UAV positioning optimization. This is performed by the backend of SLAM modules, including local mapping thread and loop closing thread. The local mapping thread updates the existing map point observations, culls the map points that are difficult to view with multiple keyframes and creates new map points. A local bundle adjustment is adopted to optimize the estimations of map points and keyframes in the local area. The keyframes that are too visually similar to the incoming keyframe will be replaced. The loop closing thread detects motion loops and corrects the pose and position estimation of keyframes to resolve the drift of long-term estimation. It is noteworthy that the tracking thread calculates on the basis of a local map. When the local map, including map points and keyframes, is changed, it not only corrects past estimations but also affects current calculations.

The second task is to detect, map and locate the semantic objects. This is performed by two parallel threads: semantic segmentation and semantic positioning (i.e., the green boxes in Figure 3). The segmentation thread is responsible for segmenting the semantic object in the keyframe image, while the semantic positioning thread restores the depth of the segmented area from successive keyframe images and locates objects. The spatial coordinates of the object are detemined by the pose and position of the keyframe and the depth of the object in the keyframe image plane. In other words, the position of the semantic object is bound to the keyframes. When the keyframes are optimized by the local mapping thread or loop closing thread, the global position of the object will be recalculated. Details of the semantic modules will be provided in Section 3.2.

The module of the map is not an actual thread, but a conceptual integration of databases distributed across modules. Figure 3 utilizes it to represent the output interface of PTI-SLAM and the visualization is only used to illustrate the output of the system. In practice, the visual interface is not deployed in order to reduce the system overhead.

It is noteworthy that PTI-SLAM conveys a loose coupling between the SLAM task and the semantics task to benefit the real-time performance. In our geolocation architecture, the positioning of the UAV is provided by the SLAM part in real time, while the semantic object positioning serves the coordinates transformation. A delay in the semantic task only causes a delay in the correction of UAV geolocation.

### 3.2. Semantic Positioning

**Figure 4:**
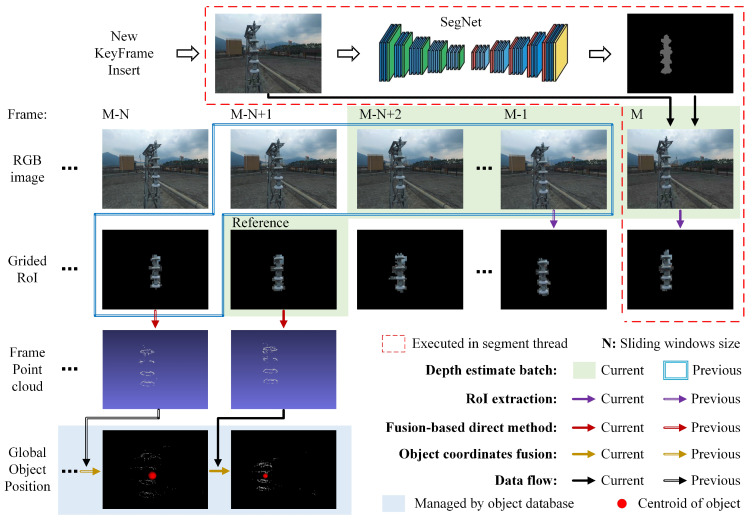
The visualization flowchart of semantic object positioning.

Figure 4 shows the overview of the semantic positioning. The semantic task can be divided into three steps: two-dimensional semantic segmentation, three-dimensional semi-dense mapping and object positioning.

Following DS-SLAM, PTI-SLAM adopts SegNet, which is a deep fully convolutional neural network architecture for semantic segmentation, as the semantic extractor to process the raw RGB images of keyframes and provide pixel-wise segmentation. Owing to the possible surface reflection, concerning the segmentation of the object, there are occasionally voids or incomplete edges.To overcome this limitation, a grid-based region of interest (RoI) strategy is designed.The grid strategy uses voxel-like ideas to simplify two-dimensional semantic representations and tolerate the internal voids caused by misdetection. More specifically, when the input images are 640×480 resolution, they are gridded in 10×10 pixels. The grids that contain semantic pixels are treated as the object region and combined into RoIs. Subsequently, those grids surrounded by RoIs are also merged, as they are considered as the misdetection. RoIs which are two grids away from each other are regarded as the independent RoI belonging to separate objects. The RoIs with a too small area are filtered, as they usually come from incorrect segmentation or overly distant objects.

As for three-dimensional semi-dense mapping and object positioning, they are performed by the proposed fusion-based semantic direct method. Instead of long-term pixel tracking, the fusion-based direct method utilizes a sliding window to establish individual batches and locally track the pixel to densely reconstruct and position the objects and then globally fuse the object position.

Let $F_{i},i=0,1,2,\cdots ,M$ denote keyframes; the newest keyframe is $F_{M}$; the object positioning based on sliding batch can be presented as follows:

(1) $$O_{ref}=\left\{O_{M-N+1,j}\right\}=f\left(F_{M-N+1},F_{M-N+2},\cdots ,F_{M}\right)$$

where $O_{M-N+1,j}$ indicates the object coordinates in the camera coordinates of the reference keyframe, *j* is the number of objects and *N* is the sliding windows size (it is empirically taken as 10 in our case; more details are provided in Section 4.5.1). $f\left(\cdot \right)$ denotes the object positioning using inverse depth filter within batch. In other words, the first keyframe in the batch is used as the reference frame; we track the pixels of the object forward in turn and densely map the object. To economise on the computational load, PTI-SLAM models the object as centroids, as the red dot shown in Figure 4. Details of the inverse depth filter and object positioning within batch are provided in Section 3.2.1 and  Section 3.2.2.

To globally fuse the object positioning, the object coordinates need to be transformed into SLAM coordinates; this can be easily obtained as follows:

(2) $$O_{ref}^{W}=T_{ref}^{-1}\cdot O_{ref}$$

where $O_{ref}^{W}$ denotes object coordinates in SLAM coordinates and $T_{ref}^{-1}$ is the transformation matrix from the reference frame to SLAM coordinates provided by SLAM part. Then, the object coordinates are fused as follows:

(3) $$\left\{\begin{array}{c} O_{fuse,J}^{W}=\frac{w_{J}\cdot O_{J}^{W}+w_{M-N+1,j}\cdot O_{M-N+1,j}^{W}}{w_{J}+w_{M-N+1,j}}\\ w_{fuse,J}=w_{J}+w_{M-N+1,j} \end{array}\right.$$

where *w* is the existing fusion weight of the object and the subscript fuse presents the updated estimates. We employ the quantity of the point cloud of the object in the reference frame as $w_{M-N+1,j}$. The subscript *J* denotes the global object associated with $O_{M-N+1,j}$. Details of the object association are provided in Section 3.2.3

The fusion-based semantic direct method is designed based on two assumptions that have been observed several times in our scenario testing:

- Although illumination changes often occur, the brightness of the image is usually consistent in a small area. By limiting the range of pixel tracking to a small region on both a spatial and a temporal scale, our method enhances the confidence in the assumption of brightness constancy.
- A few extreme cases, such as drastic but transient reflections from the target surface, can destabilize the direct method, resulting in a sharp decrease in the quantity of generated point clouds. We utilize this property to reduce the contribution of these unreliable observations to object positioning.

#### 3.2.1. Semi-Dense Mapping for RoIs

**Figure 5:**
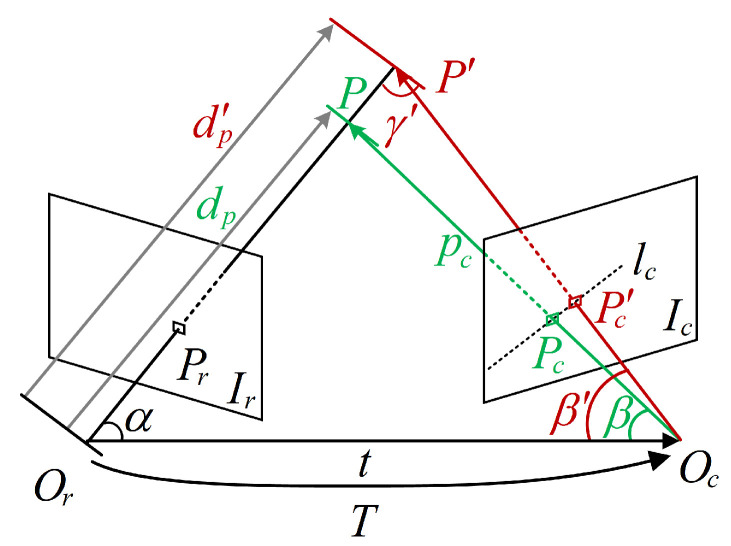
Depth estimation based on direct method.

Figure 5 shows how the direct method restores depth from two adjacent keyframes. $I_{r}$ and $I_{c}$ are the images of the reference frame and current frame, $O_{r}$ and $O_{c}$ denote the corresponding camera optical centres, $P_{r}$ and $P_{c}$ are the projection points of *P* in $I_{r}$ and $I_{c}$, respectively. According to the epipolar geometry, $P_{r}$ and $P_{c}$ are constrained to lie along conjugate epipolar lines $l_{c}$. Geometrically, $l_{c}$ is the projection of extension of $O_{r}P_{r}$; it can be uniquely determined by giving $P_{r}$ and the transformation matrix *T* of the camera. To find the match point $P_{c}$, the pixel blocks on $l_{c}$ are examined in a stepwise manner by calculating its zero-mean normalized cross correlation (ZNCC) [21] with the block of $P_{r}$:

(4) $$S{\left(A,B\right)}_{ZNCC}=\frac{\sum_{i,j}\left(A\left(i,j\right)-\bar{A}\right)\left(B\left(i,j\right)-\bar{B}\right)}{\sqrt{\sum_{i,j}{\left(A\left(i,j\right)-\bar{A}\right)}^{2}{\left(B\left(i,j\right)-\bar{B}\right)}^{2}}}$$

where $A,B\in R^{\omega \times \omega }$ are the pixel blocks in $I_{r}$ and $I_{c}$, respectively, $S{\left(A,B\right)}_{ZNCC}$ presents the matching score. $A\left(i,j\right)$ and $B\left(i,j\right)$ are the pixel values in *A* and *B*, $\bar{A}$ and $\bar{B}$ denote the mean value of the corresponding blocks. Only the block pairs with $S{\left(A,B\right)}_{ZNCC}>0.85$ will be considered as the potential match and the best of them is regarded as the correct match. By subtracting the mean pixel value, the local brightness variation of the image can be suppressed to some extent. To speed up the traversal of blocks, the search domain is defined as follows:

(5) $$\left\{\begin{array}{cc} p_{cmax}\left(X,Y,Z\right) & =\left(d_{ini}+3\sigma \right)T{\left(O_{r}P_{r}\right)}^{0}\\ p_{cmin}\left(X,Y,Z\right) & =\left(d_{ini}-3\sigma \right)T{\left(O_{r}P_{r}\right)}^{0} \end{array}\right.$$

where $p_{cmax}$ and $p_{cmin}$ are the spatial points corresponding to the endpoints of the search domain on epipolar, representing the hypothetical range of the spatial point *P* on the extension of $O_{r}P_{r}$. They are determined by depth assumption $d_{ini}$ (i.e., the hypothetical distance from *P* to image plane $I_{r}$) and depth uncertainty σ and then transfer to camera $O_{c}$ coordinate by transformation matrix *T*. Transformation matrix *T* represents the transformation relationship between the reference frame and the current frame, including the translation of the position and rotation of perspective. They are given by the SLAM module, i.e., the keyframe database in the map module. $d_{ini}$ and σ are artificially assigned at the very beginning, then automatically iteratively updated in the depth filter. $p_{cmax}$ and $p_{cmin}$ can be easily mapped into $I_{c}$ by the pinhole camera model:

(6) $$P_{c}\left(u,v\right)=\left\{\begin{array}{c} u=f_{x}\frac{X}{Z}+c_{x}\\ v=f_{y}\frac{Y}{Z}+c_{y} \end{array}\right.$$

where $f_{x}$, $f_{y}$, $c_{x}$ and $c_{y}$ are the camera intrinsics.

Estimating the depth of the pixel from only two frames is not reliable, as the baseline between keyframes is usually short. To combat this problem, the inverse depth filter is employed to fuse observations from multiple keyframes. As Figure 4 shows, the last *M* keyframes are packaged into an estimation batch, where *M* is the sliding window coefficient. In the process of a single batch, the first frame is used as the reference frame. The depth of the RoIs in the reference frame is estimated with the subsequent frames one by one using the direct method described above. There are M−1 depth estimations for reference frames in total. To fuse these observations, the estimated depths are represented as the Gaussian probability distribution over the inverse depth [22] and fused as follows:

(7) $$\left\{\begin{array}{c} \mu_{fuse}=\frac{\sigma_{obs}^{2}\mu +\sigma^{2}\mu_{obs}}{\sigma^{2}+\sigma_{obs}^{2}}\\ \sigma_{fuse}^{2}=\frac{\sigma^{2}\sigma_{obs}^{2}}{\sigma^{2}+\sigma_{obs}^{2}} \end{array}\right.$$

where μ and σ denote the existing inverse depth estimate and the error-variance used in (5), the subscript obs indicates the estimates of new incoming items and the subscript fuse presents the updated estimates. Owing to the strategies of ZNCC and sliding window, we only consider the geometric disparity error [23] in the calculation of $\sigma_{obs}$. Suppose there is a one-pixel error in the matching of $P_{r}$, as shown in Figure 5, where $P_{c}^{{}^{\prime }}$ stands for a match with error. The observation uncertainty can be obtained by:

(8) $$\sigma_{obs}=\left|\frac{1}{∥d_{p}∥}-\frac{1}{∥d_{p}^{{}^{\prime }}∥}\right|=\left|\frac{1}{d_{p}}-\frac{sin\gamma^{{}^{\prime }}}{∥t∥sin\beta^{{}^{\prime }}}\right|$$

To avoid inefficient observation, only the pixels that exceed the gradient threshold of 50 are selected for depth estimation.

At the last stage within batch processing, the depth estimates of the reference frame are filtered with respect to the threshold of σ to erase untrustworthy observations.Qualified estimates are used to generate the point cloud of the reference frame.

#### 3.2.2. Object Positioning within Batch

**Figure 6:**
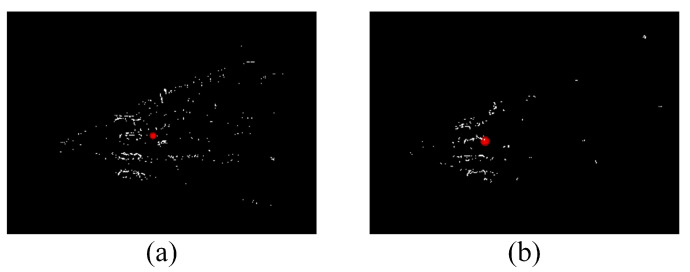
Visualization of semantic positioning process. (**a**) Unfiltered point cloud and centroid (red dot) of the RoI in single batch. (**b**) The point cloud and its centroid after statistical filtering.

Essentially, depth recovery is an estimate of the norm of $d_{p}$ in Figure 5. The point cloud of the RoI is always distributed in a pyramidal space, as shown in Figure 6 and the outliers typically come from estimation errors and background noises. Owing to the scale uncertainty of monocular SLAM, the statistical filtering method is adopted to filter out noise. More specifically, in a single RoI block, the average distance between a point and its nearest 80 points is calculated; then the mean and standard deviation of the average distances are worked out to exclude the points that exceed 0.5 times the standard deviation. After statistical filtering, the residual outliers, however they are distributed, have very little effect on the centroid because of the significant difference in numbers between inliers and outliers.

#### 3.2.3. Object Association

To speed up object association, the potential associated global object is suggested by two-dimensional reprojection and is verified by three-dimensional distance statistics. Specifically, the global centroids of objects are reprojected into the reference frame image to identify the centroids within three grids of the RoI. The qualified centroids are projected into the reference frame point cloud to calculate its average distance to the points and compare with the centroids of the reference frame. When the former one is not greater than twice the latter, the global centroid is considered as the same object as the reference frame centroid.

Objects in the reference frame that cannot be matched by the object database are temporarily recorded as potential new objects. A new object can only be established if it can be observed in three consecutive batches. Such a mechanism can reject occasional fake observations.

### 3.3. Object-Based Geographical UAV Positioning

The proposed strategy can be described as follows (Algorithm 1):
**Algorithm 1:**Object-based geographical UAV positioning**Input:** UAV position and pose of reference frame in SLAM $UP_{Sr}$,       UAV position and pose of current frame in SLAM $UP_{Sc}$,       object position of reference frame in SLAM $OP_{Sr}$,       ENU coordinate of object $OP_{E}$**Ouput:** UAV position and pose of current frame in ENU coordinates $UP_{Ec}$,  1:**while** observed objects ≥3 **do**  2:   Given $OP_{E}$ of these objects  3:   Performs umeyama algorithm [24] $umeyama\left(OP_{Sr},OP_{E}\right)$ to obtain the rotation matrix *R*, translation vector *t* and scale factor *s*  4:   Performs sim3 transformation: $UP_{Er}=\langle R,s\times UP_{Sr}\rangle +t$  5:   **if** continuous observation ≥12 **then**  6:     Find the five observations with the lowest object positioning deviation to the current fusion result, obtain their $UP_{Sri}$ and $UP_{Eri}$  7:     $R_{gol},t_{gol},s_{gol}=umeyama\left(UP_{Sri},UP_{Eri}\right)$  8:     $UP_{Ec}=\langle R_{gol},s_{gol}\times UP_{Sc}\rangle +t_{gol}$  9:   **end if**10:**end while**11:**while** observed objects ≤3 **do**12:   **if** $R_{gol}$, $t_{gol}$ and $s_{gol}$ are empty **then**13:     Waiting for initialization14:   **else**15:     $UP_{Ec}=\langle R_{gol},s_{gol}\times UP_{Sc}\rangle +t_{gol}$16:   **end if**27:**end while**

The essence of the strategy can be simply summarised as:

- When the observation of objects is possible, obtain the transformation matrix and recalculate the UAV position by using the object associations between SLAM and the GPS.
- When objects are not observed, transform the UAV positioning in the SLAM coordinates into GPS coordinates using the existing transformation matrix.

It is noteworthy that the Umeyama algorithm [24] adopted in Algorithm 1 needs at least three matched object pairs to calculate the transformation variables. In practice, it is not difficult to find multiple semantic objects (such as insulator, damper and other potential large-size fittings) for the hover points.

## 4. Experimental Results

### 4.1. Environment Setup

**Figure 7:**
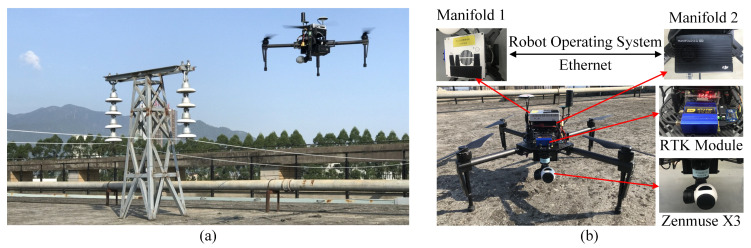
The experiment scene and inspection UAV. (**a**) Simulated transmission tower scenario. (**b**) The prototype of the inspection UAV.

All experiments in this paper are carried out in the simulated transmission tower scene, as shown in Figure 7a. The test site is set up on the roof to create an open scene with a high view resembling the actual UAV view. The transmission tower is represented by a miniature ’T’ type tower model assembled with the suspended ceramic insulator strings and link fittings of a 35 kV power tower. The circumvolant flight path proposed in [7] is adopted in place of a typical flight scheme as it can perform a flight distance similar to the circular inspection of the 35 kV power tower.

To verify the performance in the onboard system, PTI-SLAM is deployed in the prototype of the inspection UAV. As Figure 7b shows, the prototype is a refitted DJI Matric 100 quad-rotor UAV equiped with a pan-tilt camera Zenmuse X3, two advanced low-power-consumption embedded processors—DJI Mainfold 1 and DJI Mainfold 2-GPU—and an additional RTK module. While Mainfold 1 takes charge of communicating with the sensors and the flight controller, Mainfold 2-GPU, with more computing power, is primarily responsible for running algorithms. These two scattered subsystems are connected by a local area network and communicate via a robot operating system (ROS). The groundtruths, including the UAV trajectories and the locations of insulators, are obtained with the RTK module, with an absolute error of less than 2 centimeters.

The images captured by the Zenmuse X3 camera are resized to 640×480 resolution and then fed into PTI-SLAM. PTI-SLAM employs ORB-SLAM2 as the backbone of the SLAM part for monocular UAV positioning; 1500 point features for image at eight scale levels with a scale factor of 1.2 are extracted in ORB-SLAM2. As for the semantic part, the sliding window coefficient *M* is taken as 10. The initial values of $d_{ini}$ and σ are empirically set to 3 and 0.1, respectively. Meanwhile, the threshold of σ for erasing the untrustworthy observations in the generation of the frame point cloud is 0.001.

### 4.2. Trajectory Consistency Evaluation

The trajectory consistency represents the ability to keep track of UAV position during continuous movement, which plays a significant role in UAV flight control. For example, the autopilot in the DJI onboard software development kit (SDK) is realized by controlling the deviation between the current position and the destination, all of which are represented in local coordinates taking the take-off point as the origin. In other words, the performance of autonomous flight is determined by the tracking accuracy of continuous motion relative to the origin, rather than the absolute positioning accuracy of the GPS. In this part, the trajectory consistency of PTI-SLAM is tested and compared with airborne GPS to explore the feasibility of PTI-SLAM for UAV inspection flight control. A comparison of existing methods is provided in Section 4.6. Concerning quantitative assessment, the absolute trajectory error (ATE), which stands for the global consistency of the trajectory, is adopted and the metrics, including root mean squared error (RMSE), mean error, max error, min error and standard deviation (S.D.), are presented in Table 2, where x, y and z items show the errors along the x-y-z axes of the east north up (ENU) coordinate system and ’Resultant’ denotes the resultant errors.

**Figure 8:**
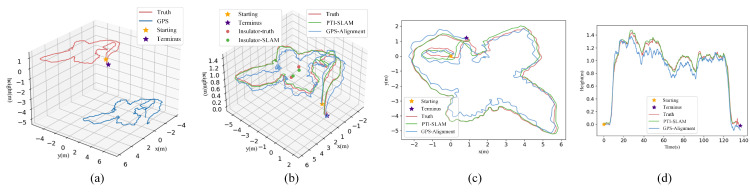
The trajectory comparison of PTI-SLAM and GPS. (**a**) Trajectory drawn by airborne GPS. (**b**) Trajectory comparison of the proposed PTI-SLAM and GPS-alignment in 3D view. (**c**,**d**) Horizontal and vertical components of trajectories.

In practical terms, civilian GPS usually has an error of 2–15 m. As other airborne sensing data are fused by the SDK, our airborne GPS draws a relatively accurate trajectory during continuous motion and the positioning error often manifests as the shift of the origin point, as shown in Figure 8a. While the RMSE and mean error of GPS nearly reach 8 m, the S.D. of GPS is only 0.2, signifying that the error likely comes from a global consistent offset. For further comparison, the zero of GPS trajectory is aligned with the true value, that is, the GPS-alignment, which has an RMSE and mean error of only 0.37 and 0.35 m, respectively, and a max error of no more than 0.65 m.

As illustrated in Figure 8b–d, the trajectory estimated by the proposed PTI-SLAM is more closely matched to the groundtruth than the GPS-alignment, both in the horizontal (x-y axes) and vertical (z axis) components. As seen in the last item of Table 2, the proposed PTI-SLAM has an overall advantage over airborne GPS, with errors less than half those of the GPS-alignment. This advantage of motion estimation demonstrates the high potential of PTI-SLAM for UAV inspection.

### 4.3. Evaluation of Object Positioning

**Figure 9:**
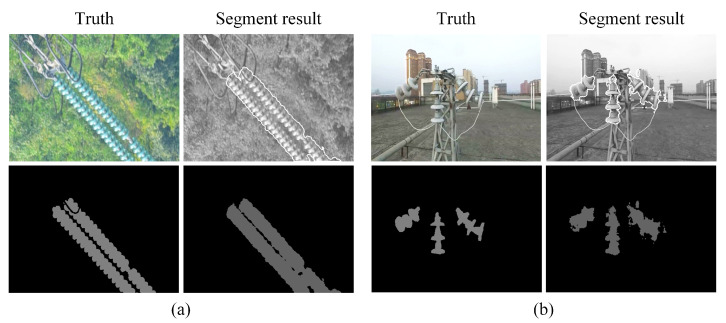
The examples of segmentation results. (**a**) The segmentation result on actual inspection. (**b**) The segmentation result on simulated scene.

SegNet is trained using an actual inspection dataset with 3741 images and tested with 418 images. After 56,000 iterations of training, it achieves a result of 92.3% mean pixel accuracy (MPA) and 88.6% mean intersection over union (MIoU). Figure 9 shows the visualization of segmentation results on actual inspection and a simulated scene.

The positioning for an object is estimated on the basis of the positions and poses of a camera given by the SLAM part. The experiment results of this part therefore incorporate the factor of the SLAM module error. Table 3 gives the effect of point cloud filtering for a single batch. For intuitive comparison, the point cloud is converted to the global coordinate system. About a quarter of the points are culled after statistical filtering. As shown in Figure 6, the culled points are mainly outliers and the errors of the centroid are significantly reduced by 39.9%.

The experiments of batch fusion are presented in Table 4. The data contains a total of 21 valid keyframes coming from a 14 second flight period of simulated checkpoint examination. The observation distance is within the range of 1–3.8 m. By setting the sliding window coefficient *M* to 10, there are 12 estimate batches in total.

The semantic positioning module delivers a competitive object positioning effect with RMSEs of no more than 0.4 m. The errors and S.D. along the x axis are notably larger compared to the others. This is mainly due to the error distribution of SLAM positioning. As seen in Table 2, the SLAM error in the x axis is about one-third larger than in the y axis, which is similar to the error distribution of object positioning. Altogether, the object positionings for a complete flight around the tower are visualised in Figure 8b.

### 4.4. Evaluation of Object-Based Geographical Positioning

To satisfy the requirement of the Umeyama algorithm, two additional insulators are added to each side of the tower model. Note that the object association between SLAM coordinates and geographical coordinates is created artificially. In fact, establishing this association mathematically is an urgent issue that needs to be addressed in a follow-up. In addition, the incomplete object observations (i.e., the RoI of the object is located on the image boundary) are rejected for object positioning.

**Figure 10:**
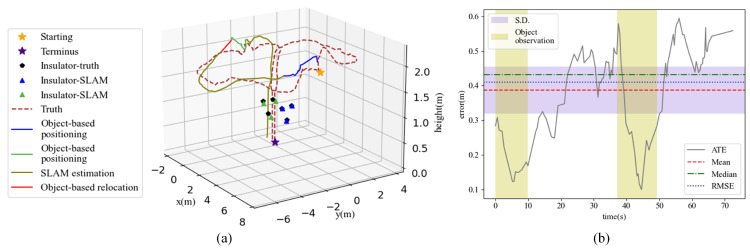
Object-based geographical UAV positioning. (**a**) The visualization of object-based UAV positioning. (**b**) The error statistics.

Figure 10 shows the results of object-based UAV repositioning. In Figure 10a, we denote the SLAM trajectory with several colors. The blue and green segments indicate the intervals with the observations of blue and green insulators, respectively. They are located by the dynamic transformation variables $R_{gol}$, $t_{gol}$ and $s_{gol}$. The olive segment represent the interval without object observation and is estimated by the fixed transformation variables. The red line is the correction for cumulative error of the olive segment.

As seen in Figure 10b, the RMSE and mean error are about 0.4 m and the max error is up to 0.6 m. The ATEs are not uniformly distributed along the trajectory, resulting in a notably greater S.D. compared to the PTI-SLAM. While the ATE is generally on the rise without object observation, there is a noticeable error reduction around the object observation intervals. This suggests that the object observations can significantly reduce the positioning error and the positioning accuracy can be improved by adding more scattered objects.

### 4.5. Study on Object Positioning Performance

#### 4.5.1. Sliding Window Size

Table 5 shows the tests on sliding window coefficient *N*. In a normal scene, each of the two additional frames in the batch takes an extra 0.7 s to process and increase the number of point cloud, leading to finer details concerning the object modelling but with little effect on object positioning. However, increasing *N* allows more candidate frames into the batch, enhancing the success rate of the direct method in disadvantageous scenarios. An extremely disadvantageous scenario concerning examining the target against the sun is set up to test this idea. We deliberately move the UAV from side to side, facing the sunlight to check the target and the brightness of the image changes due to the drastic change in the lighting conditions. In such an adverse situation, the object cannot be located only by a few adjacent keyframes, as shown in Table 5; the object positioning is invalid when N=4. By adding more candidate frames, the direct method can find more pixel matching from the later frames and give a decent object positioning with a low fusion weight (i.e., the number of point clouds).

For some extreme cases, such as the first observation of semantic object being in an extreme backlight condition, this strategy can provide a rough positioning at the beginning and then gradually correct it in subsequent observations. Otherwise, it takes another 5–10 s to establish a reliable object positioning. In order to ensure the adaptability of PTI-SLAM to these unfavorable conditions, the value of *N* is the maximum value allowed by the computation time. For PTI-SLAM in testing scene, the keyframe generation period is about 0.656 s, so we set N=10.

#### 4.5.2. Influence Factor

In practice, there are three main factors that affect object positioning accuracy: direct method error, SLAM module error and background noise. Direct method errors are mainly caused by a mismatch of image block pairs. According to Figure 5, the mismatch of block pairs will only lead to an incorrect depth estimate on spatial points and the error is then assigned to each axis of the coordinate according to the view angle. SLAM module errors, including camera position and orientation, not only cause direct method errors (since they can affect the determination of the epipolar lines), but also lead to errors in coordinate transformation. Background noise is driven by segmentation accuracy and is intensified by the grid strategy. It usually appears at the rear of the target because the UAV typically observes the objects from the outside, resulting in a backward deviation of object positioning, as seen in Figure 6.

**Figure 11:**
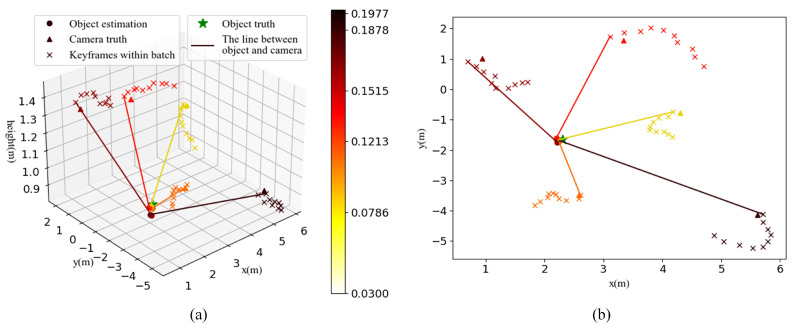
Multiple view object positioning evaluation. (**a**) Three-dimensional view. (**b**) Overhead view.

In order to investigate the factors influencing object positioning, an assessment was conducted with movement around the east side of the tower. To stably control the motion variables, the data in this experiment are acquired with a hand-held UAV. Figure 11 shows five representative object positionings, which are estimated from different angles and distances. The results are denoted in different colors according to their error in depth estimation. It is noteworthy that the observation results are the raw data without batch fusion. The marker × represents the locations of the keyframes within the estimate batch; the marker at the end point of the line connecting the camera and the object is the reference frame.

**Figure 12:**
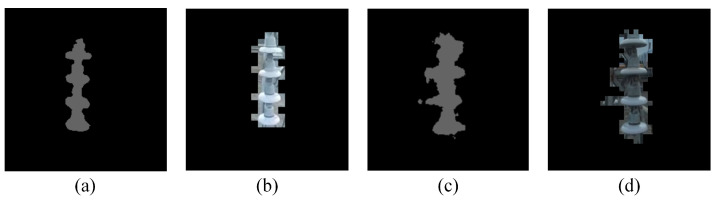
The difference between manual segmentation and automatic segmentation. (**a**,**b**) The example of manual segmentation and its gridded RoI. (**c**,**d**) The example of automatic segmentation and its gridded RoI.

Table 6 shows the quantitative results. The object positioning is evaluated in two metrics: depth error and absolute positioning error (APE). The depth error is the deviation in the distance between the object and the camera, which is mainly affected by the matching error and excludes the effect of coordinate transformation. The APE is the position deviation between estimated object location and the truth, representing the comprehensive error level. We adopt the angle between $l_{est}$ and $l_{gt}$ to approximately express the orientation error of SLAM, where $l_{est}$ is the line connecting the estimated object and the estimated camera and $l_{gt}$ is the line connecting the groundtruths of the object and the camera. To eliminate the influence of the difference in segmentation results, the objects are manually segmented in this experiment. The difference between manual segmentation and automatic segmentation is shown in Figure 12. Three additional factors, which most likely contribute to the observation error, are examined: the distance from the camera to the object, the object location in the image and the movement range within the batch. The object location in the image is denoted by the deviation between the object pixel centroid and the image center. We numbered the observations according to their error in depth estimation.

As seen in Figure 11 and Table 6, the depth estimate error is not directly related to the viewing angle, the SLAM error and the movement range within the batch, but has a strongly positive relationship with the object distance and the object location in the image. This is consistent with the previous information:

- The accuracy of visual measurement decreases with increasing distance and the measurement for the object at the edge of the view field is usually less accurate than in the central area. The latter is partly related to lens distortion, even if distortion correction has been performed.
- The fusion-based direct method uses the relative relationships of a position and orientation between frames, rather than the absolute positioning of each frame, to restore the depth. In a short-term gradual motion, SLAM typically performs good movement tracking capabilities and the estimation errors of relative orientation and position between frames tend to converge to a consistent range. This error is less related to the absolute error of the current frame, unless the batch happens to be in an extremely unfavourable situation. Therefore, in normal conditions, there is no direct correlation between the positioning error of the camera and the depth estimation error.

The absolute location of the object is essentially the projection of the depth estimation onto the global map. Theoretically, the APE is positively correlated with the depth error and the SLAM error. According to Table 6, the orientation error of SLAM has a much greater impact on APE than the location error of SLAM and the depth error and even dominates. When the orientation errors are similar, such as obs-1 and obs-5, the depth error has a greater impact on the object positioning than the positioning error of SLAM.

Compared with Table 4, the object positioning errors in Table 6 are significantly lower, indicating that the background noise plays an essential role in the object positioning error. This means that the proposed method is sensitive to the background noise and highly dependent on noise filtering.

In term of overall performance, the positioning of the object can converge in a small range, which ensures the reliability of the batch fusion. As shown in Figure 11, the depth estimate is slightly greater than the truth. This is mainly caused by the background noise. The gridding strategy improves the recall of 2D segmentation while also increasing the background noise of observations.

### 4.6. Comparison of Methods

#### 4.6.1. Robustness of Algorithms

To verify the advancement, the proposed PTI-SLAM is compared with other iconic methods which are widely used as the baselines in many studies. The proposed PTI-SLAM shares a consistent configuration with ORB-SLAM2 to guarantee a fair comparison. The direct ORB-SLAM 2, which is a restructuring ORB-SLAM2 accelerated by the direct method of SVO [25], extracts the features twice as much as PTI-SLAM to improve its robustness in a testing scene. We also compared the LSD-SLAM, which is a milestone in direct method-based SLAM and is adopted as a SLAM part in a monocular semantic SLAM [11].

All the methods are compared in three different operation scenes: smooth flight in steady illumination (termed the normal scene), flight with occasional rapid rotation in steady illumination and smooth flight in variable illumination. Two datasets are collected for each scene. The methods are tested five times separately on each dataset and the tracking success rate and average RMSE of ATE are presented in Table 7. As to the tracking success rate, there are two situations that can be judged as tracking failure. The first one is that the system state is lost and the relocation fails.The second case is where the RMSE is too large (more than 0.8 meters in this paper); the algorithm can be considered to have actually failed.

Direct methods, including direct ORB-SLAM2 and LSD-SLAM, have a significantly lower success rate than feature-based SLAM. By adopting a more advanced depth filter, direct ORB-SLAM2 is notably more accurate than LSD-SLAM; however, the success rate is not improved. In contrast, the feature-based SLAM demonstrates a high robustness against disadvantageous scenes, especially illumination variation. As a result of using the feature-based method in the SLAM part, the success rate and accuracy of PTI-SLAM are similar to ORB-SLAM2, indicating that PTI-SLAM inherits the robustness advantage of the feature-based approach. It is noteworthy that PTI-SLAM is slightly less accurate than ORB-SLAM2. This is mainly due to the fact that semantic tasks take up computational resources and reduce the executions for backend optimization. Although PTI-SLAM adopts the direct method in semantic modules, its robustness is not compromised.

**Figure 13:**
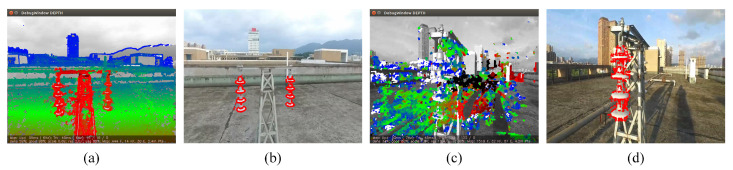
A comparison of the traditional direct method and the proposed fusion-based direct method. (**a**) Dense mapping of LSD-SLAM in stable scene. (**c**) Dense mapping of LSD-SLAM under adverse conditions. (**b**,**d**) Dense mapping for semantic object using fusion-based direct method.

Figure 13 shows a comparison of the traditional direct method, which is represented by LSD-SLAM and the fusion-based direct method. The red dots in Figure 13b,d represent the pixels with filtered pointcloud. In the normal scene, the fusion-based direct method creates a dense mapping similar to the LSD-SLAM. In the scene of intense lighting, there is a strong reflection on the surface of the tower and the insulator, resulting in a failure of LSD-SLAM. In contrast, the fusion-based direct method still works as expected.

#### 4.6.2. Time Consumption

For time analysis, the time consumptions for each module are tested and the results are listed in Table 8. DS-SLAM [20] is taken as the baseline because it shares the same algorithms with PTI-SLAM for 2D semantic extraction and SLAM solving. The tracking thread is the main source of the real-time requirement as it is demanded to respond in a timely manner to the image input and provide UAV positioning. The direct methods have significant advantages over feature-based SLAM in time consumption. Even so, the processing speed of exceeding 12 frames per second of ORB-SLAM2 also satisfies the real-time requirements [7]. As for semantic SLAM, DS-SLAM serially performs a semantic task for every frame in a tracking thread, resulting in low tracking efficiency A tracking speed of 2 frames per second makes practical applications on airborne platforms impossible. By stripping the semantic-related tasks, the track thread of PTI-SLAM is executed at a speed exceeding 12 frame per second, which is consistent with the original ORB-SLAM2. Compared with Cube-SLAM, which is an advanced monocular object SLAM and is also improved from ORB-SLAM2, PTI-SLAM shows a significant improvement in tracking speed, demonstrating its advantage in real-time performance.

As for the time consumption on semantic tasks, there are two parallel threads in PTI-SLAM responsible for the semantic task. In the segment thread, the keyframe can be handled in 0.29 s, while the average period of keyframe generation is 0.645 s; hence, the requirements of keyframe segmentation can be easily satisfied. The time consumption of the semantic positioning thread is related to the RoI size of reference frame. When taking the whole image as RoI, the processing time can be up to 2.31 s. Table 9 represents the average time consumptions of semantic positioning threads in the presence and absence of RoI, which are 0.7 s and approximately zero, respectively. Attributed to the parallelism of threads, the incoming keyframe can be segmented during the object positioning, so the overall elapsed time for the semantic positioning is still 0.7 s. As shown in Figure 2, there are a large number of non-RoI flight areas during the whole inspection, in which the time consumption of object positioning is approximately zero. Therefore, all the keyframes can be processed even though the time consumption of semantic positioning is slightly larger than the generation of keyframes.

In our geographical UAV positioning strategy, the processing speed of semantic positioning only affects the refresh rate of geographical positioning correction, for which a delay of seconds is not fatal.

#### 4.6.3. Geographical Positioning Comparison

Table 9 shows the comparisons on geographical UAV positioning of PTI-SLAM and the existing visual method for tower inspection. The artificial operating performance reported by the China Southern Power Grid (CSG) [28] is adopted as the baseline. Generally, the hovering error of artificial operation is about 1.5 m, indicating that the inspection target can be observed within this error range. This provides a good reference baseline to verify the feasibility of our approach.

The UGA-UAV cooperative architecture [8] mentioned in the introduction reports the max positioning errors of 1.75 and 2.07 m at different resolutions of input image without using RTK in UAV. By taking tower components as the positioning identifier, our approach reduces the measurement distance to a small range and presents a significant accuracy gain.

## 5. Discussion

Power tower inspection is a serial task oriented by scene structure. Since some scene structures are difficult to locate directly, considering the visual navigation as structure-based geolocating and geographical waypoint flight is a feasible scheme at present. By using semantic SLAM, geolocating can be converted into estimating the transformation between SLAM and geographic coordinate systems based on semantic landmarks. This scheme can reduce real-time requirements for semantic tasks. Once the system has completed the initialization of geolocation, the delay of the semantic task will only affect the refresh rate of geolocation correction. Taking this advantage, the proposed PTI-SLAM removes semantic tasks from the real-time constraints by loosely coupling semantic tasks and SLAM tasks. In the time consumption comparison, we verify that PTI-SLAM can achieve an additional semantic task without slowing down the tracking speed of the SLAM part. This scheme can be seamlessly plugged into the current inspection works and can be combined with the study of tower-structure-based automatic waypoint planning [29] to further explore the possibility of autonomous UAV tower inspection. In addition, its idea can be easily extended to other similar applications.

The challenge of the scene mainly comes from the complex and changeable lighting conditions. The proposed fusion-based direct method utilizes a sliding window strategy to address this challenge and realize semantic semi-dense mapping. The sliding window strategy is essentially a fault tolerance mechanism. For the light changes to continue as fragments, it enhances the confidence in the assumption of brightness constancy by limiting the range of pixel tracking to a small region on both a spatial and a temporal scale. For the transient changes to a few frames, it increases the fault tolerance to local variation by adding more alternative frames for the pixel tracking. In the SLAM part, the robustness experiment preliminarily proves that the feature-based SLAM approach can effectively deal with this challenge; however, it still has a risk of failure during fast rotation. According to previous studies [30], the fusing of the camera and the IMU can alleviate this problem and also facilitate the restoration of the monocular scale. Since there is a transition between the gimbal camera and the body, this is a challenging information fusion problem of three systems with different sampling frequencies: the camera, the IMU and the gimbal attitude sensing.

As for the performance of semantic object positioning, there are two additional influence factors besides the accuracy of segmentation and the SLAM part that should be noted: the distance between the object and the camera and the offset between the object and the image center. The former suggests that the distance factor can be taken into account in the fusion of object observation, while the latter indicates that some optimization aid, such as auxiliary aiming and shooting, can be considered. Generally, we regard our approach as a rough global positioning solution of guiding UAV to approach the inspection target and a finer adjustment for data acquisition should be achieved by an auxiliary shooting subsystem.

## 6. Conclusions

This paper reports an investigation on using the structure of a power tower to realize visual positioning of UAV for tower inspection and presents a monocular semantic positioning framework to cope with the challenge of a scene. To offer advice on semantic selection, the potential of the tower component as the semantic object for inspection flight is examined in terms of scene structure describing ability and visual detection adaptability. The insulator and damper are strong semantic candidates, while some of the large-size fittings and ancillary facilities, such as clamp and tower plate, are of great promise but need more evidence to prove their visual detection adaptability. The proposed PTI-SLAM conveys a hybrid architecture combining the feature-based SLAM method with the direct method to juggle positioning robustness and mapping density. A fusion-based direct method is presented to improve the robustness of the direct method against the adverse conditions in the inspection scene by limiting the pixel track and taking semantic observation fusion as a substitute in global tracking. The trajectory consistency evaluation shows that PTI-SLAM offers a better motion estimate compared to the airborne GPS, verifying its feasibility for UAV control. Comparisons of methods demonstrate that the hybrid architecture inherits the advantages of both the feature-based method and the direct method. The loose coupling between the SLAM task and the semantics task guarantees real-time performance and preserves the robustness against a complex scene with the assistance of a fusion-based direct method. To explore the application of PTI-SLAM in tower inspection, an object-based geographical UAV positioning strategy is devised. The preliminary experiment indicates more competitive accuracy compared to the artificial UAV operation and the previous visual approach, proving the potential of PTI-SLAM for inspection.

Compared with the existing UAV positioning approaches, PTI-SLAM breaks the dependence on GPS and provides additional environment information, producing the prospect of autonomous UAV inspection. In future works, establishing additional constraints of semantic mapping may contribute to richer semantic modelling, thereby improving semantic map accuracy and providing more possibilities for semantic applications. In general, this work presents the first part of a more intelligent transmission tower inspection system. There are certain other aspects that must be investigated to practically implement autonomous inspection, such as object association between SLAM and the actual world, dynamic path planning based on object observation and obstacle avoidance.

## Figures and Tables

**Table 1:** The developments of visual detection studies on tower inspection aerial images.

Objects	Object Detection	Aerial Image Fault Detection (mAP)	
	Literature	UAV	Copter
Foundation	/	44%	56%
Cable	Precision: 94% [17]	66%	23%
Insulator	AP: 96.4% [18]	74%	89%
Damper	AP: 95.2% [19]	/	87%
Large-size fittings	mAP: 78.6% [15]	77%	/
Small-size fittings	/	87%	67%
Tower	AP: 95% [16]	63%	/
Ancillary facilities	AP: 73.2% [16]	52%	82%

**Table 2:** The comparison of absolute trajectory error (meters).

Method	Component	RMSE	Mean	Median	Max	Min	S.D.
GPS	Resultant	7.7874	7.7847	7.8375	8.1800	7.2905	0.2065
	x	1.5370	1.5254	1.5160	1.9826	1.1034	0.1885
	y	5.4175	5.4095	5.4410	5.9644	4.7497	0.2936
	z	5.3788	5.3784	5.3839	5.5642	5.1226	0.0696
GPS-alignment	Resultant	0.3790	0.3523	0.3790	0.6457	0.0003	0.1399
	x	0.2010	0.1629	0.1594	0.5271	0.0001	0.1177
	y	0.3053	0.2602	0.2711	0.6383	0.0001	0.1597
	z	0.1004	0.0855	0.0820	0.2581	0.0003	0.0526
PTI-SLAM	**Resultant**	**0.1447**	**0.1219**	**0.1058**	**0.2693**	**0.0085**	**0.0779**
	x	0.1192	0.0921	0.0798	0.2119	0.0009	0.0757
	y	0.0787	0.0598	0.0448	0.1803	0.0005	0.0512
	z	0.0230	0.0176	0.0129	0.0515	0.0003	0.0148

**Table 3:** Point cloud filtering in single batch.

	Centroid (m)			Point Cloud Number
	x	y	z	
Unfiltered	1.4361	−1.8895	0.8450	7295
Statistical filtered	1.1414	−1.8834	0.8489	5514
Groundtruth	0.7759	−2.0697	0.8904	/

**Table 4:** Object positioning based on multi-frame fusion.

	Centroid (m)			Euclidean Distance Error (m)
	x	y	z	
Groundtruth	0.7759	−2.0697	0.8904	/
1st batch	0.9977	−1.9386	0.8331	0.2639
2 batches fusion	0.9765	−1.8928	0.8471	0.2709
4 batches fusion	0.9827	−1.9155	0.8457	0.2618
6 batches fusion	1.0182	−1.9001	0.8418	0.2997
8 batches fusion	1.0627	−1.8784	0.8445	0.3478
10 batches fusion	1.0552	−1.8942	0.8447	0.3319
12 batches fusion	1.0538	−1.9164	0.8542	0.3194
RMSE	0.2472	0.1656	0.0463	0.3011
Mean	0.2449	0.1645	0.0460	0.2993
Median	0.2423	0.1695	0.0457	0.2997
S.D.	0.0336	0.0184	0.0059	0.0322

**Table 5:** Testing on sliding window size.

Testing Scene	Window Coefficient *N*	Object Location Error (m)	Time (mSec)			Point Cloud Number
			Total Delay	Depth Estimation	Positioning	
Normal	6	0.2855	521	488	33	4270
	8	0.2842	601	567	34	4437
	10	0.2845	671	637	34	4537
	12	0.2834	740	706	34	4626
	15	0.2856	829	795	34	4687
Extreme disadvantage	6	**failed**	402	401	1	/
	8	0.5479	579	575	4	388
	10	0.5514	702	696	6	801
	12	0.5625	849	842	7	948
	15	0.6049	958	950	8	1114

**Table 6:** The error statistics for multiple view object positioning.

	Object Error		SLAM Error		Object Distance (m)	Deviation from Center (pixel)	Mean Movement of Keyframes (m)
	Depth	Location	Location	Orientation			
obs-1	0.0786	0.0791	0.0176	1.9693	2.224	49.6688	0.8462
obs-2	0.1009	0.1213	0.0009	3.2653	1.8644	73.8296	0.845
obs-3	0.1515	0.1118	0.0662	2.3923	3.443	109.0948	1.8682
obs-4	0.1878	0.143	0.0724	3.0785	3.0254	145.2951	1.2238
obs-5	0.1977	0.1108	0.0107	1.9836	4.1482	79.1419	1.0855

**Table 7:** Comparison on robustness of algorithms.

Method	Attributes	Tesing Scene	Success Ratio	RMSE (m)
LSD-SLAM [26]	direct method dense mapping	normal	6/10	0.3731
		rapid rotation	4/10	0.4126
		light change	3/10	0.4335
Direct ORB-SLAM2	direct method semi-dense mapping	normal	7/10	0.0911
		rapid rotation	3/10	0.2232
		light change	3/10	0.2159
ORB-SLAM2 [27]	feature-based sparse mapping	normal	10/10	0.1083
		rapid rotation	6/10	0.2332
		light change	10/10	0.197
PTI-SLAM	hybrid method sparse environment semi-dense object	normal	10/10	0.1279
		rapid rotation	6/10	0.2802
		light change	10/10	0.2150

**Table 8:** Time consumption comparison of different algorithms.

Method	Attributes	Runtime (mSec)		
		Track	Semantic Segmentation	Semantic Positioning
LSD-SLAM [26]	monocular direct method	42	/	/
Direct ORB-SLAM2	monocular direct method	49	/	/
ORB-SLAM2 [27]	monocular feature-based	81	/	/
DS-SLAM2 [20]	RGB-Depth/semantic feature-based	506	298	/
Cube-SLAM [13]	monocular/semantic feature-based	130	/	/
PTI-SLAM	monocular/semantic hybrid method	81	291	703/0

**Table 9:** Comparison of geographical UAV positioning.

		RMSE	Mean	Max	Min	S.D.
Our		0.4094	0.3865	0.5952	0.0989	0.1351
[8]	640×368	/	0.45	2.07	/	0.30
	1280×720	/	0.25	1.75	/	0.28
Artificial flight [28]		1.50	1.50	1.76	1.26	0.111

## Data Availability

The authors do not have permission to share data.

## Abbreviations

The following abbreviations are used in this manuscript:

UAV	Unmanned Aerial Vehicle
SLAM	Simultaneous Localization And Mapping
PTI-SLAM	Power Tower Inspection SLAM
RTK GPS	Real-Time Kinematic fixed Global Positioning System
LiDAR	Light Detection And Ranging
RGB-D	RGB-Depth
RMSE	Root Mean Squared Error
ATE	Absolute Trajectory Error
RoI	Region of Interesting
AP	Average Precision
CEPRI	China Electric Power Research Institute
ZNCC	zero-mean normalized cross correlation
ROS	Robot Operating System
SDK	Software Development Kit
MAP	Mean Pixel Accuracy
MIoU	Mean Intersection over Union
**Symbols**
$F_{i}$	The i-th keyframe
$O_{ref}$	The set of the coordinates of the object of reference frame,
	in the coordinate system of reference frame
$O_{M,j}$	The coordinate of the j-th object of keyframe M,
	in the coordinate system of reference frame
f(·)	Object positioning using inverse depth filter
$O_{ref}^{W}$	The set of the coordinates of the object of reference frame,
	in the coordinate system of SLAM
$T_{ref}$	The transformation from SLAM coordinates to reference frame
$O_{J}^{W}$	The existing coordinate of object J in SLAM coordinate system
$O_{M,j}^{W}$	The coordinate of the j-th object of reference frame,
	in the coordinate system of SLAM
$O_{fuse,J}^{W}$	The updated coordinate of object J in SLAM coordinate system
$w_{J}$	The existing fusion weight of object J
$w_{M,j}$	The fusion weight of the j-th object of reference frame
$w_{fuse,J}$	The updated fusion weight of object J
$I_{r}$	The image of the reference frame
$I_{c}$	The image of the current frame
$O_{r}$	The camera optical centres of the reference frame
$O_{c}$	The camera optical centres of the current frame
*P*	The spatial point

$P_{r}$	The projection points of *P* in $I_{r}$
$P_{c}$	The projection points of *P* in $I_{c}$
$l_{c}$	The epipolar lines of *P* in $I_{c}$
$S{\left(A,B\right)}_{ZNCC}$	The matching score obtained by ZNCC
A,B	the pixel blocks in $I_{r}$ and $I_{c}$ respectively
A(i,j),B(i,j)	The values of the pixels in *A* and *B*
$\bar{A},\bar{B}$	the mean value of *A* and *B*
$p_{c}max\left(X,Y,Z\right)$	The hypothetical max depth of the spatial point *P* to $I_{c}$
$p_{c}min\left(X,Y,Z\right)$	The hypothetical min depth of the spatial point *P* to $I_{c}$
$d_{ini}$	The initial value of depth estimating
μ	The expected value of inverse depth of *P* in the Gaussian distribution
σ	The error-variance of Gaussian distribution,
	it can be used as depth uncertainty
$P_{c}\left(u,v\right)$	The coordinate of the projection of *P* in $I_{c}$
$f_{x},f_{y},c_{x},c_{y}$	The camera intrinsics
$\mu_{fuse}$	The updated inverse depth estimating
$\sigma_{fuse}$	The updated error-variance
$\mu_{obs}$	The inverse depth estimating of new incoming
$\sigma_{obs}$	The error-variance of new incoming
$d_{p}$	The true depth of *P* in $I_{r}$
$d_{p}^{{}^{\prime }}$	The false depth of *P* in $I_{r}$
$UP_{Sr}$	UAV position and pose of reference frame in SLAM coordinate system
$UP_{Sc}$	UAV position and pose of current frame in SLAM coordinate system
$OP_{Sr}$	object position of reference frame in SLAM coordinate system
$OP_{E}$	object position of reference frame in SLAM coordinate system
$UP_{Ec}$	UAV position and pose of current frame in ENU coordinate system
*R*	The rotation matrix
*t*	The translation vector
*s*	The scale factor
